# Milk-derived extracellular vesicles: nature’s nanocarriers for drug delivery and therapeutics

**DOI:** 10.3389/fphar.2025.1595891

**Published:** 2025-08-06

**Authors:** Chen Kong, Long-bin Huang, Mei-feng Yang, Ning-ning Yue, Yuan Zhang, Cheng-mei Tian, Yuan-hui Wang, Dao-ru Wei, Rui-yue Shi, Yu-jie Liang, Jun Yao, Li-sheng Wang, De-feng Li

**Affiliations:** ^1^ Department of Gastroenterology, Shenzhen People’s Hospital, The Second Clinical Medical College, Jinan University, Shenzhen, Guangdong, China; ^2^ Department of General Medicine, Yantian District People’s Hospital, Shenzhen, Guangdong, China; ^3^ Department of Medical Administration, Huizhou Institute of Occupational Diseases Control and Prevention, Huizhou, Guangdong, China; ^4^ Department of Emergency, Shenzhen People’s Hospital (The Second Clinical Medical College, Jinan University, The First Affiliated Hospital, Southern University of Science and Technology), Shenzhen, Guangdong, China; ^5^ College of Rehabilitation Medicine, Jining Medical University, Jining, Shandong, China; ^6^ Department of Rehabilitation, Shenzhen People’s Hospital (The Second Clinical Medical College, Jinan University, The First Affiliated Hospital, Southern University of Science and Technology), Shenzhen, Guangdong, China; ^7^ Department of Child and Adolescent Psychiatry, Shenzhen Kangning Hospital, Shenzhen Mental Health Center, Shenzhen, Guangdong, China; ^8^ Department of Gastroenterology, Shenzhen People’s Hospital (The Second Clinical Medical College, Jinan University, The First Affiliated Hospital, Southern University of Science and Technology), Shenzhen, Guangdong, China

**Keywords:** milk, extracellular vesicles, targeted therapy, exosomes, drug delivery

## Abstract

Breast milk-derived extracellular vesicles (MEVs) are natural nanocarriers characterized by their stability, biocompatibility, and low immunogenicity. These small, lipid bilayer-enclosed nanoparticles carry diverse bioactive molecules, including proteins, nucleic acids, and lipids, enabling them to facilitate inter-organismal communication. This review highlights the therapeutic potential of MEVs as innovative drug delivery systems, with a focus on their unique composition, functional properties, and mechanisms of action—from biogenesis and secretion to cellular uptake. We critically examine current methods for isolating and purifying MEVs, addressing challenges related to scalability, purity, cost, and standardization in industrial production. Furthermore, we discuss strategies to enhance the bioavailability and stability of MEVs for pharmaceutical applications. In conclusion, MEVs represent a scalable and cost-effective platform for therapeutic delivery, with significant potential in both nutritional and medicinal contexts. Future research should focus on optimizing production processes and advancing clinical translation to fully harness their capabilities.

## Graphical Abstract

**Figure:**
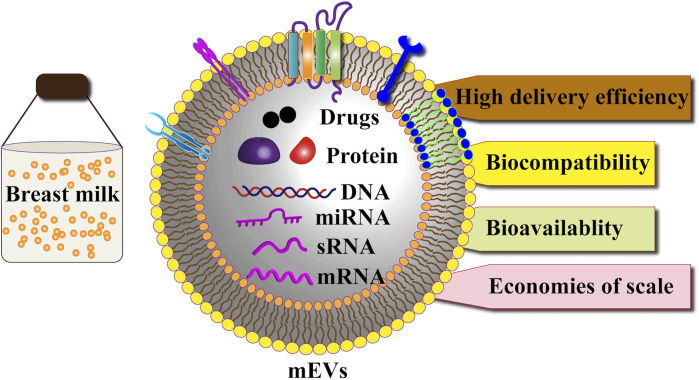
MEVs are a promising therapeutic tool.

## 1 Introduction

Extracellular vesicles (EVs) are lipid bilayer-enclosed particles released from cells that lack the ability to replicate independently (Vaiaki and Falasca, 2024). They carry proteins, lipids, and nucleic acids, facilitating intercellular communication and pathophysiology (Shen et al., 2022; Pishavar et al., 2022). Encased in a phospholipid bilayer, EVs resist degradation in harsh gastrointestinal tract conditions (Li et al., 2022a; Zhu et al., 2023). Furthermore, EVs have several advantages (Li et al., 2022a; Cheng et al., 2023; Durmaz et al., 2024). First, their high biocompatibility, low immunogenicity, and lack of toxicity *in vivo* make them promising drug delivery carriers. Second, their double-layered lipid membranes protect therapeutically relevant molecules in the gastrointestinal tract and enable long-term circulation in the body. Third, exosomal membranes can be artificially modified for targeted binding to specific organs and cells, making them promising therapeutic agents in clinical practice. Previously, we reviewed the biogenesis, characteristics, composition, functions, and drug delivery potential of EVs derived from plants, gut microbiota, mesenchymal stem cells, and immune cells in diagnosing and treating gastrointestinal diseases (Li et al., 2022a; Li et al., 2023a; Tian et al., 2023a; Tian et al., 2023b; Liu et al., 2023a; Li et al., 2022b).

**FIGURE 1:**
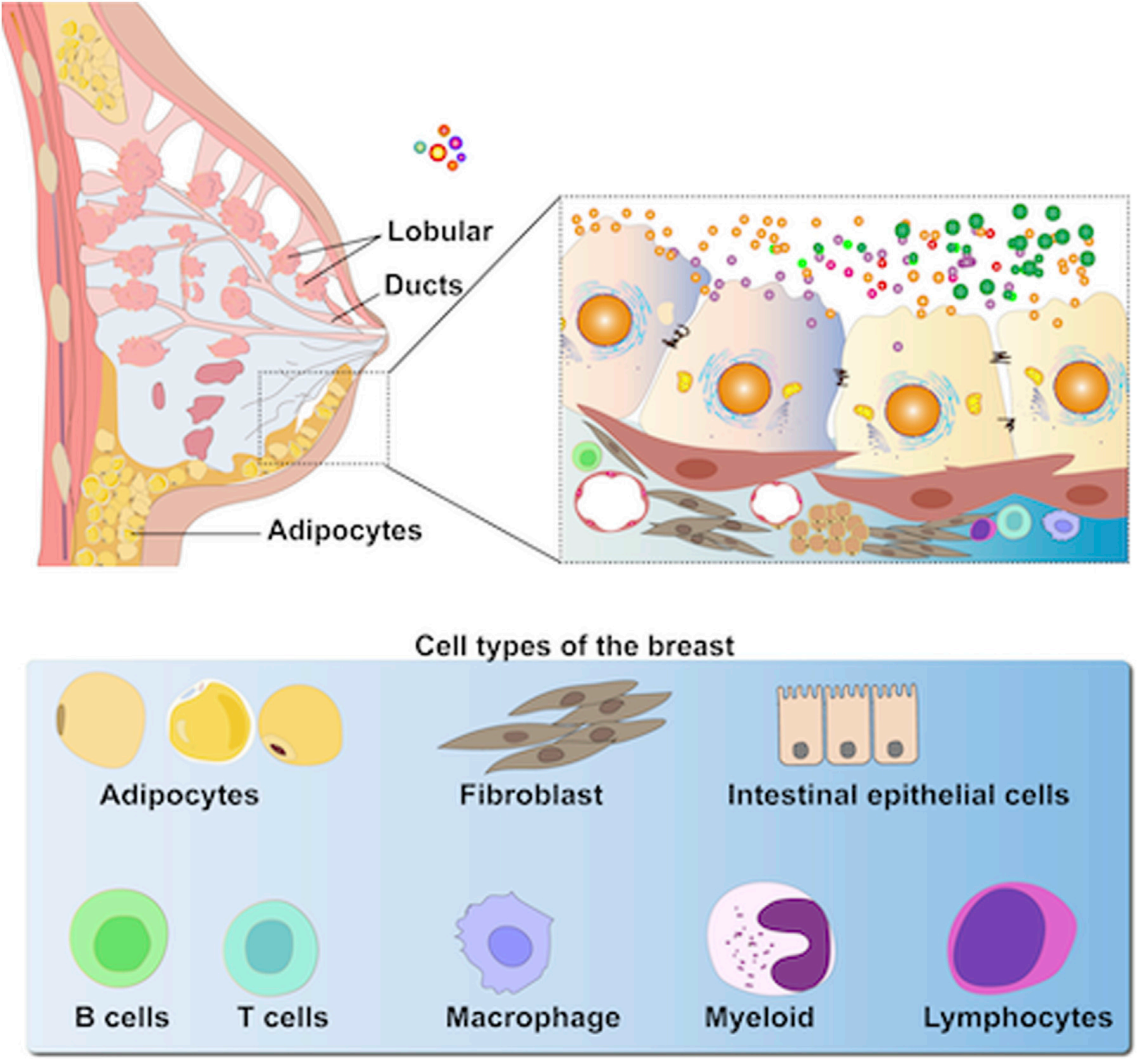
MEVs are highly heterogeneous as they are secreted primarily by epithelial cells within mammary alveoli and stored in the lumen of alveoli adjacent to these cells (including immune cells, stem cells, bacteria, and adipocytes, etc.).

Mammalian milk, a heterogeneous fluid that provides nutrition to newborn mammals, contains several bioactive components, such as proteins, antibodies, and peptides that modulate the immune system modulation, promote cell growth, and exhibit antioxidant effects (Einerhand et al., 2022; Liu et al., 2021; Hobbs et al., 2021). Figure 1 shows the pathway of EVs from the breast tissue to milk. Recently, milk-derived EVs (MEVs) from sources such as humans, goat, and sheep have garnered significant attention for their therapeutic potential in human metabolism, immunology, and physiology. Moreover, MEVs have great potential as drug delivery vehicles and in imaging and therapeutic applications owing to their non-toxicity, high availability, low immunogenicity, and stability (Kim et al., 2023; Timofeeva et al., 2023; Tian et al., 2023c; Li et al., 2022c; Garcia-Martinez et al., 2022). This review explores MEVs, detailing their isolation, purification methods, and therapeutic applications, and summarizes their bioavailability, biocompatibility, and immunogenicity in drug delivery systems. Additionally, the challenges for industrializing of MEV production are highlighted.

## 2 Characteristics of MEVs

MEVs share common components with other EVs, including Alix, Flillin1, transmembrane proteins (CD9, CD63, and CD81), integrins, and cell adhesion molecules, while also containing unique proteins, such as testilin, Rab GTPase, and Tsg101, which regulate membrane fusion, interact with cytoskeletal proteins, and participate in endocytosis (van Herwijnen et al., 2016). Moreover, MEVs have specific markers for butyrin, lactase, and xanthine dehydrogenase, and offer unique advantages over other EVs (Vaswani et al., 2021). For instance, resistant glycoproteins (XDH, BTN, and MUC1) encapsulating MEVs and their surface proteins (FLOT1, ICAM1, ALIX, and EpCAM) enhance their resistance to pepsin and ensure stability in the gastrointestinal tract (Benmoussa et al., 2019). Furthermore, MEVs contain mRNAs, microRNAs (miRNAs), and DNAs transferred to offspring through breastfeeding, playing vital roles in infant development, including gastrointestinal structure and function, bone metabolism, endocrine regulation, and metabolism. Beyond their physiological roles, MEVs can cross the intestinal mucosal barrier and enter the bloodstream, enhancing the oral bioavailability of protein and several small-molecule drugs while reducing dosage and toxicity compared to cytotoxic anticancer drugs alone. This section explores the biogenesis and cargo of MEVs.

### 2.1 MEV biogenesis

**FIGURE 2:**
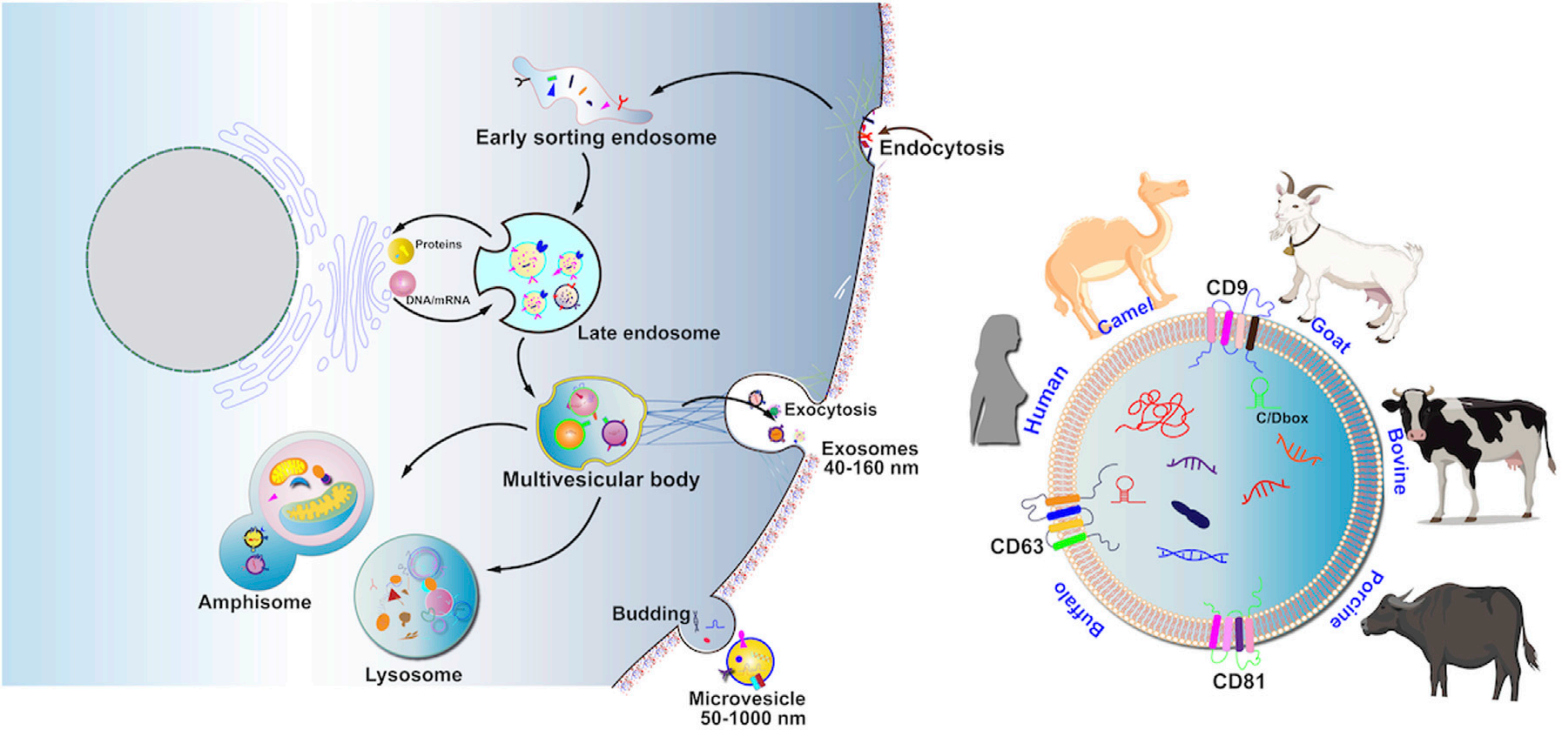
MEV production involves four processes including cargo sorting, MVB formation, maturation and transportation of MVB, and fusion of MVB with the cell membrane. Recent research-based articles reported on MEVVs in the animal kingdom, including cattle, humans, pigs, goats, donkeys, buffaloes, and camels.

EV biogenesis begins with endocytosis of invaginated endosomes from the plasma membrane, predominantly involving both endosomal sorting complex required for transport (ESCRT)-dependent and ESCRT-independent machinery (Figure 2). The detailed process of EV biogenesis is available in our previous reviews (Li et al., 2022a; Li et al., 2023a; Li et al., 2022b).

### 2.2 Cargoes in MEVs

MEV cargoes primarily contain proteins, lipids, and nucleic acids, with variations depending on the origin of the MEV cells, individual differences, and physiological conditions. These cargoes of MEVs are delivered to recipient cells, where they carry out physiological functions and biological actions. This section reviews the functions and biological actions of MEV cargoes.

Proteome analysis of MEVs has been used to detect over 2000 proteins, which vary across different MEVs and proteomic methods. For example, more than 2000 proteins have been detected in bovine MEVs, whereas 1963 proteins have been identified in human MEVs (van Herwijnen et al., 2016; Reinhardt et al., 2012). MEV proteins are categorized into specific and functional proteins (Table 1). Specific proteins, initially identified in human and bovine MEVs, have not been detected in EVs derived from other biofluids or cell types and may serve as potential MEV biomarkers. Van Herwijnen et al identified 1963 proteins in human MEVs, of which 633 were exclusive to MEV. Bioinformatics analysis revealed that these proteins are significantly enriched in cytoskeletal/structural activity, transmembrane receptor protein tyrosine kinase activity, and cell adhesion (van Herwijnen et al., 2016). Admyre et al demonstrate that specific MEV proteins can inhibit anti-CD3-induced cytokine production in peripheral blood mononuclear cells and increase the number of Foxp3^+^CD4^+^CD25^+^ T regulatory cells. However, the specific proteins identified are currently limited. (Admyre et al., 2007). Moreover, bovine MEVs contain unique adhesion proteins such as intercellular cell adhesion molecule-1, surface receptors, and glycoproteins that play vital roles in signal transduction pathways and molecular (including drug) delivery (Pullan et al., 2022; Wehbe and Kreydiyyeh, 2022). MEVs also carry several functional proteins involved in cell growth, tissue regeneration, immune modulation, and drug delivery. However, few functional proteins have been identified, and this area requires further investigation.

**TABLE 1:** The proteins in the MEVs.

Proteins biomolecule	Biological function	References
MHC class II, CD81, MUC-1, heat shock proteins and lactadherin	Cell binding, several enzymes, and cytosolic components	Admyre et al. (2007)
1963 unique proteins identified, such as tetraspanins CD9, CD63, CD81, annexins and Ras-related proteins	Cytoskeletal/structural activity, transmembrane receptor protein tyrosine kinase activity, and cell adhesion	van Herwijnen et al. (2016)
239 proteins identified, such as lactadherin, Perilipin-2, Butyrophilin, Xanthine Oxidase, tetraspannins, CD9, and CD81	Binging, catalytic activity, molecular adaptor activity, etc.	Vaswani et al. (2021)
227 proteins identified, such as butyrophilin, Xanthine oxidase/dehydrogenase, Adipophilin (perilipin 2), and Lactadherin	Binging, catalytic activity, molecular adaptor activity, molecular transducer activity, etc.	Vaswani et al. (2021)
2107 proteins identified, such as butyrophilin, Xanthine oxidase, Adipophilin, Lactadherin	Antioxidant, binding, catalytic, etc.	Reinhardt et al. (2012)
1372 proteins identified, a lot of marker proteins were detected, such as Alix and TSG101	Bioinformatic analysis showed these proteins were involved in immune response regulation and growth	Samuel et al. (2017)
2350 proteins identified,	Defense response, defense response to bacterium, and response to bacterium	Reinhardt et al. (2013)
1838 proteins identified, including XDH, BTN1A1 and MFGE8	Bioinformatic analysis showed these were mainly involved in translation regulation, protein maturation and cell structural maintenance.	Benmoussa et al. (2019)
2225 proteins identified,	Bioinformatic analysis indicated that these proteins were engaged in mammary gland physiology, milk production, immunity, and immune response.	Rahman et al. (2021)
639 proteins identified	Bioinformatical analysis showed these proteins were enriched in acute inflammatory response, complement activation, classical pathway, B cell mediated immunity, negative regulation of blood coagulation and coagulation, activation of immune response and protein maturation and processing.	Chen et al. (2017)
637 proteins identified	Endothelial cell development and lipid metabolism	Grossen et al. (2021)
CD81, CD63, serum albumin, lactoferrin, lactadherin, beta-lactoglobulin, kappa-casein, kappa-casein precursor, beta-casein, alpha-S1-casein, and alpha-S1-casein precursor	Immunomodulation and cell growth regulation, antibody-dependent cytotoxicity, cytokine production, lipid peroxidation induction, and natural killer cells activation	Sedykh et al. (2017)

MEVs contain lipid molecules embedded in their membrane structure, including phosphatidylcholine, phosphatidylethanolamine, and phosphatidylserine, which maintain their structure and stability while facilitating cargo transport to recipient cells (Table 2) (Grossen et al., 2021). Moreover, bioactive lipids in MEVs are crucial for gastrointestinal health, supporting neonatal intestinal development, protecting the intestinal epithelium, and inhibiting intestinal inflammation. Studies on the lipid bioactivities of MEVs are relatively scarce. Lipidomic profiling has identified 395 lipids in human MEVs (Chen et al., 2021). Additionally, the top 50 lipids significantly decrease necrotizing enterocolitis (NEC) severity and alleviate intestinal epithelial cell damage by inhibiting the ERK/MAPK pathway activity (Chen et al., 2021). However, current technology faces challenges in isolating and identifying lipids from MEVs (Ong et al., 2021; Blans et al., 2017).

**TABLE 2:** The lipids in the MEVs.

Lipids biomolecule	Biological function	References
Sphingomyelin, phosphatidylcholine, phosphatidylethanolamine and phosphatidylserine	n/a	Blans et al. (2017)
Phospholipid, Sphingomyelin, phosphatidylcholine	n/a	Blans et al. (2017)
Phosphatidylcholine (PC), phosphatidylserine (PS), and phosphatidylethanolamine (PE)	Decreasing the severity of necrotizing enterocolitis (NEC), and alleviated the damage of intestinal epithelial cells	Chen et al. (2021)

Besides their protein and lipid profiles, MEVs contain several nucleic acids, including mRNAs, microRNAs, and double-stranded DNAs. Studies show that these nucleic acids contribute to their anti-inflammatory and immunomodulatory activities (Table 3). Microarray analyses have revealed 19,320 mRNAs in bovine MEVs (Izumi et al., 2015). Moreover, bovine MEVs are taken up by human macrophages (THP-1 cells), promoting their differentiation through cargo mRNAs (Izumi et al., 2015). Another study identified 16,304 mRNAs, including 13,895 known and 2,409 novel mRNAs, in porcine MEVs (Chen et al., 2017). Moreover, bioinformatics analysis indicates that most of these mRNAs are mainly involved in binding activities such as nuclear hormone receptor and protein kinase functions and diverse enzymatic activities, including transcription coactivators, exonucleases, and small conjugating protein ligases (Chen et al., 2017). Ma et al found that miR-3168, enriched in breast MEVs, plays a crucial role in early neurodevelopment and neural stem cell differentiation in preterm infants (Ma et al., 2024). Gao et al discovered that miR-30a-5p, a key component of coat MEVs, ameliorates the intestinal epithelial cell-6 inflammatory response and it attenuates lipopolysaccharide (LPS)-induced intestinal inflammation (Gao et al., 2024). However, research on DNA function in MEVs is scarce. Table 3 highlights a few nucleic acids and their biological functions in MEVs, offering just a glimpse into this area of research. Therefore, future studies should explore this promising field.

**TABLE 3:** The nucleic acid in the MEVs.

Nucleic acid	Biological function	References
1523 miRNAs identified	Bioinformatic analysis showed these miRNAs play a potential role in endocrine signaling, cellular community, and neurodevelopment	Kupsco et al. (2021)
55 lncRNAs identified, which of them CRNDE, DANCR, GAS5, HOTAIRM1, NCBP2-AS2, OIP5-AS1, PRKCQ-AS1, SNHG8, SRA1, TUG1, and ZFAS1 detected more than 50%, and CRNDE, DANCR, GAS5, SRA1, and ZFAS1detected more than 90%.	n/a	Karlsson et al. (2016)
MiR-148a-3p, miR-22-3p, miR-30d-5p, let-7b-5p and miR-200a-3p detected	Chromatin and chromosome organization, transcription and negative regulation of gene expression and bio-synthetic processes	Simpson et al. (2015)
309 miRNAs identified	Bioinformatical analysis revealed that these miRNAs were involved in cell growth and/or maintenance, cell communication and signal transduction	van Herwijnen et al. (2018)
200 miRNAs identified	Regulating gene expression.	Benmoussa et al. (2020)
15,011 circRNAs identified	Antigen processing and presentation, phagosome, protein processing in endoplasmic reticulum, endocytosis and regulation of actin cytoskeleton	Ma et al. (2021)
75 mRNA identified	Immunoglobulin domain, immunoglobulin subtype 2, immunoglobulin like fold, immunoglobulin like domain, IGc2, extracellular, and glycoprotein	Shao et al. (2018)
602 miRNAs identified	endogenous immune regulation	Kandimalla et al. (2021)
27 miRNAs identified	Immune modulation effect	Patel et al. (2019)
19320 mRNA and 79 miRNAs identified	Regulating macrophages immune response	Izumi et al. (2015)
417 miRNAs identified	Regulating immune response	Sun et al. (2015)
16304 mRNA identified	nuclear hormone receptor, protein kinase and diverse enzymatic activity	Chen et al. (2017)
69 miRNAs identified	Regulating glucocorticoid receptor signaling and neurotrophic factor mediated TRK receptor signaling	Colitti et al. (2019)

### 2.3 The biological functions of MEVs

Beyond their established biological roles, MEVs exhibit emerging therapeutic functions. They mediate epigenetic regulation by delivering miRNAs (e.g., human milk miR-148a suppresses DNMT1 in colorectal cancer cells) and circRNAs (e.g., porcine milk circ-XPO4 enhances intestinal IgA via miR-221-5p inhibition) (Kosaka et al., 2010; Zhou et al., 2021; Zeng et al., 2021; Samuel et al., 2021; Melnik and Schmitz, 2017). MEVs also modulate metabolic homeostasis, as bovine milk exosomes restore short-chain fatty acids (acetate/butyrate) and L-arginine while downregulating pro-inflammatory lipids in colitis models (Du et al., 2023). Additionally, they reshape gut microbiota composition, increasing *Akkermansia* abundance and butyrate-producing bacteria (e.g., Lachnospiraceae) to reinforce intestinal barrier integrity (Tong et al., 2021; Du et al., 2022). Crucially, MEVs facilitate maternal-infant communication by transferring immune-related miRNAs (e.g., miR-320/375 in colostrum) and circRNAs that activate VEGF signaling to promote neonatal intestinal development (Kosaka et al., 2010; Zhou et al., 2021).

## 3 Isolation and purification of MEVs

**FIGURE 3:**
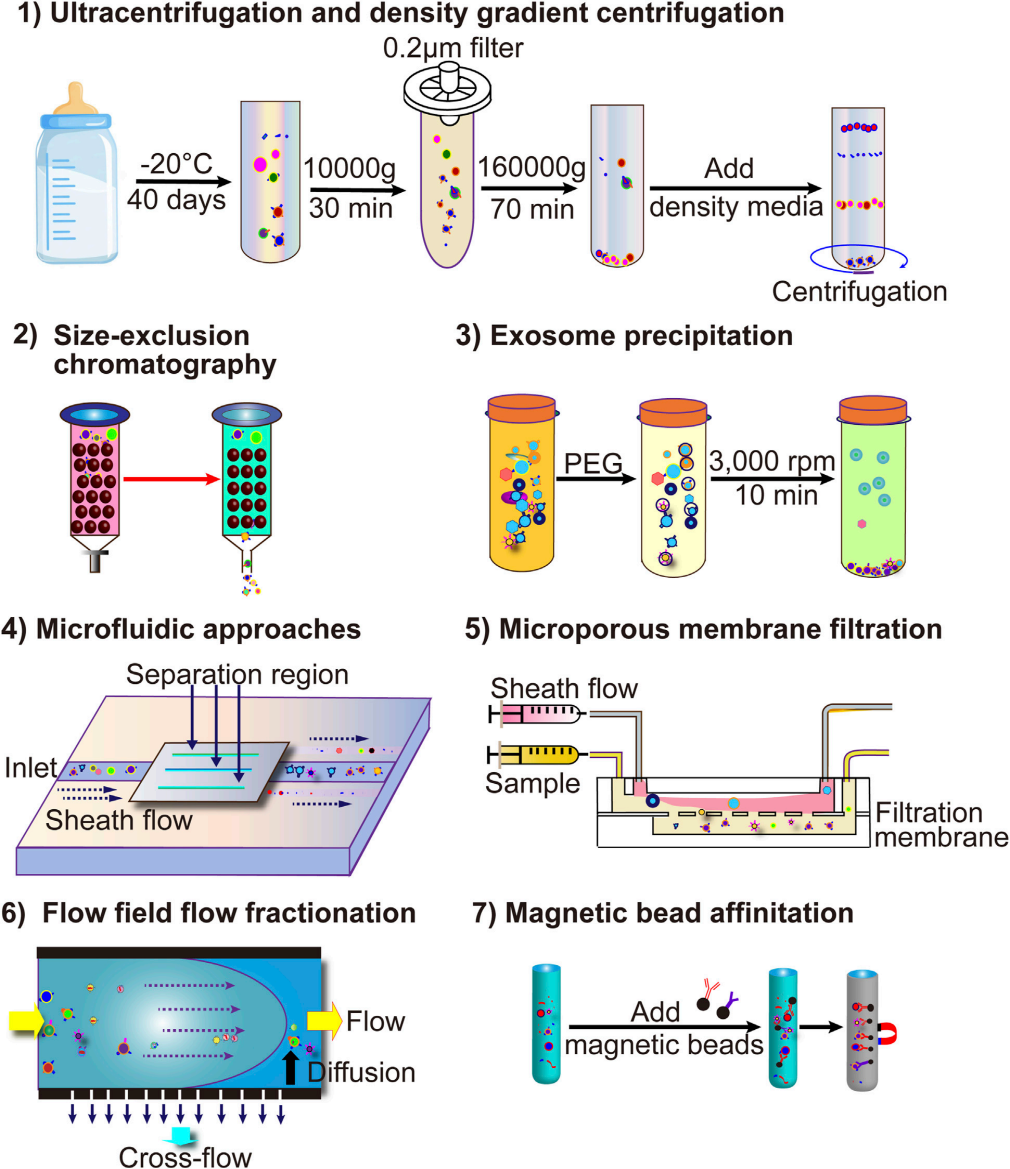
The current more common techniques for MEV isolation include ultracentrifugation, ultrafiltration, immunoaffinity capture, polymer co-precipitation, and microfluidic technology-based EV separation methods.

The complex composition of milk complicates MEV isolation and purification. Methods such as ultracentrifugation, size-exclusion chromatography, nanofiltration, immunoaffinity, and polymer-based precipitation are used to isolate and purify MEVs based on their different physicochemical properties. However, no standardized and efficient technique exists for this process. Table 4; Figure 3 present the advantages and disadvantages of various methods.

**TABLE 4:** Various methods of MEVs isolation and purification.

Method	Purity	Advantages	Disadvantages
Ultracentrifugation	Low	Simple operation, high yield, large sample capacity	Time-consuming, low purity, potential structural damage to MEVs
Size-exclusion chromatography (SEC)	High	High purity, preserves vesicle integrity, good reproducibility	Long processing time, difficult to scale up for large yields
Nanofiltration	Low	Fast processing, simple operation, minimal equipment	Risk of contamination, high EV loss, potential vesicle damage
Immunoaffinity capture	High	High specificity, simple operation, minimal equipment	Antibody-dependent, high cost, low efficiency
Polymer-based precipitation	Low	Readily available reagents, low cost, simple equipment	Low isolation efficiency, poor purification quality
Density gradient centrifugation	High	High efficiency, excellent purity, preserves integrity	High cost, complex procedure, time-consuming

### 3.1 Ultracentrifugation

Ultracentrifugation, a common technique for isolating MEVs, involves differential centrifugation (low-speed centrifugation combined with high-speed centrifugation) of milk samples to remove cell debris and free proteins, yielding purified MEVs (Thery et al., 2006). Although ultracentrifugation is effective for obtaining large quantities, it is time-consuming, highly non-specific, and damages MEVs (Table 4) (Zhong et al., 2021).

### 3.2 Size-exclusion chromatography (SEC)

SEC is an important technique for isolating MEVs based on the pore size of the stationary phase relative to EV size (Sidhom et al., 2020). As milk samples containing EVs pass through the gel column, substances smaller than the pore size are trapped, while others are removed (Sidhom et al., 2020). SEC offers several advantages for MEV isolation and purification, including high purity, integrity, and repeatability (Sidhom et al., 2020; Jia et al., 2022). However, its long running time and limited scalability are drawbacks (Table 4) (Sidhom et al., 2020; Jia et al., 2022).

### 3.3 Nanofiltration

Nanofiltration, commonly performed to isolate and purify MEVs based on EV size using the same principle as SEC, (Haraszti et al., 2018), offers several advantages, such as shorter running time, easy operation, and simple equipment. However, it is prone to contamination, EV loss, and damage (Table 4) (Haraszti et al., 2018).

### 3.4 Immunoaffinity

Immunoaffinity is a technique used to isolate and purify MEVs based on the interaction between EV surface membrane antigens and monoclonal antibodies. Specific markers such as CD9, CD63, CD81, HSP70, and TSG101 are present on EV surfaces (Thery et al., 2002; Mathivanan and Simpson, 2009; Li Y. et al., 2023; Vaswani et al., 2019; Ross et al., 2021). Therefore, specific antibodies, including anti-CD9, anti-CD63, anti-CD81, anti-HSP70, and anti-TSG10, employ immunoaffinity activity and capture targeted EVs (Sedykh et al., 2022). This method offers high specificity, ease of operation, and simple equipment. However, it is limited by marker dependence, high cost, and low efficiency (Table 4) (Shao et al., 2018).

### 3.5 Polymer-based precipitation

Polymer-based precipitation, a commonly used method for extracting MEVs, reduces EV solubility by interacting with water molecules, causing precipitation (Kandimalla et al., 2021a). Compared to other isolation methods, it is easy to use, inexpensive, and requires simple equipment. However, it has low isolation efficiency and poorly purifies MEVs (Table 4) (Patel et al., 2019; Szatanek et al., 2017).

### 3.6 Density gradient centrifugation

Density gradient centrifugation enriches EVs based on the sedimentation coefficients of different substances (Hata et al., 2010). We previously reported that sucrose gradient centrifugation effectively extracts plant-derived EV nanoparticles (Zhu et al., 2023). This method offers greater efficiency, higher purity, and better integrity than the other techniques (Szatanek et al., 2015). However, it is expensive, complex, and time-consuming (Table 4) (Szatanek et al., 2015).

Various techniques for isolating and purifying MEVs, based on different principles, have distinct advantages and disadvantages, making standardization difficult. Combining SEC with ultracentrifugation can enhance EV enrichment, purity, and integrity (Vaswani et al., 2017). We anticipate that simpler and more efficient methods or various commercial separation kits will be developed for broader clinical applications.

### 3.7 Recent advances in scalable isolation

Recent protocol optimizations demonstrate promising pathways toward GMP-compatible manufacturing of MEVs. Enzymatic pretreatment with microbial rennet (0.5% vol/vol, 37°C, 20 min) effectively eliminates casein contaminants while preserving vesicular integrity, coupled with dual centrifugation (3,000×g) for lipid removal and pre-processing freezing at −80°C to enhance purity. Subsequent isolation via differential centrifugation—scalable to tangential flow filtration—yields MEVs suitable for therapeutic applications. Critical quality assessment requires nanoparticle tracking analysis for dimensional profiling, ExoView SP-IRIS for tetraspanin validation, and Western blot for residual casein detection (Medel-Martinez et al., 2024). Various techniques for isolating and purifying MEVs, based on different principles, have distinct advantages and disadvantages, making standardization difficult. Combining SEC with ultracentrifugation can enhance EV enrichment, purity, and integrity (Medel-Martinez et al., 2024).

Current challenges in the scalable production of MEVs involve multifaceted obstacles. Industrial requirements for purity and yield fundamentally diverge from laboratory research paradigms, where the absence of standardized separation/purification methods remains the primary technical bottleneck for clinical translation—existing technologies struggle to simultaneously ensure exosome preparation homogeneity and optimized purification efficiency (Zhong et al., 2021). Further complicating the landscape are regulatory and commercialization barriers: undefined safety thresholds for interspecies applications (e.g., bovine-to-human), lack of global contaminant standards, and quality control complexities arising from source-dependent heterogeneity, compounded by inadequate regulatory frameworks for commercialization. Additionally, shortages of skilled professionals in industrial-scale exosome processing exacerbate these challenges (Zhong et al., 2021). To address these constraints, an integrated approach is essential: advancing localized implementation of ISEV characterization guidelines to establish technical benchmarks, strengthening regulatory collaboration to define safety and contaminant limits, developing scalable separation platforms (e.g., tangential flow filtration kits), and implementing specialized training programs (Medel-Martinez et al., 2024). This comprehensive strategy will systematically bridge the gap between laboratory research and industrial-scale manufacturing, thus enabling a sustainable pathway toward clinical adoption (Medel-Martinez et al., 2024). Table 5 systematically compares key characteristics of MEVs with other widely studied extracellular vesicles—mesenchymal stem cell-derived EVs and cancer-derived EVs—highlighting critical challenges in scalability, stability, targeting capability, and ethical considerations for clinical translation.

**TABLE 5:** Systematic comparison of extracellular vesicles form different sources.

	Milk-Derived Extracellular Vesicles (MEVs)	MSC-Derived Extracellular Vesicles (MSC-EVs)	Cancer-Derived Extracellular Vesicles (Cancer- EVs)
Production Yield	Scalability Challenges: - Industrial scaling limited by purity-yield trade-offs; lacks standardized methods (Zhong et al., 2021) - Enzymatic pretreatment (microbial rennet) + centrifugation improves purity; cryo-pretreatment/TFF require optimization (Medel-Martinez et al., 2024)	Inconsistent Dosing: - Clinical trials use variable units (μg, particles, cell counts) (Ebrahim et al., 2018, Sengupta et al., 2020, Lotfy et al., 2023) - Dominated by lab-scale methods (ultracentrifugation/TFF); no industrial protocols (Busatto et al., 2018, Lotfy et al., 2023)	High Secretion: - Single cancer cell releases >20,000 vesicles in 48 h (Balaj et al., 2011) - Higher abundance in biofluids than CTCs (Castro-Giner and Aceto, 2020)
Biostability	GI Resilience: - Sphingomyelin/cholesterol-rich bilayer resists gastric acid/enzymes (Théry et al., 2009; Wu et al., 2022) - Surface proteins (XDH, BTN, MUC1) enhance pepsin resistance (Benmoussa et al., 2019)	Transient Efficacy: - Systemic distribution via IV/inhalation/local delivery (Lotfy et al., 2023) - Short therapeutic duration (2–6 months) (Lotfy et al., 2023)	Extreme Biofluid Stability: - Tolerates freeze-thaw cycles & 2-day storage at 25°C (Enderle et al., 2015) - RNA stable >25 years at -80°C. - Vesicle structure protects cargo (Valadi et al., 2007, Castellanos-Rizaldos et al., 2018)
Targeting Capability	Engineered & Natural: - Integrins enable gut tropism (Warren et al., 2021) - FA/HA modification boosts tumor targeting (e.g., 74% suppression) (Munagala et al., 2016, Li et al., 2020, Cui et al., 2023) - pH-responsive drug release (hydrazone bonds) (Counihan et al., 2018, Liu et al., 2019, Zhang et al., 2020) Limitation: Species-specific affinity gaps (Zhong et al., 2021)	Disease-Driven Homing: - Intranasal delivery crosses blood brain barrier(BBB) (Lotfy et al., 2023) - Loaded therapeutics (e.g., *KrasG12D* siRNA, miR-124) enhance efficacy.	Biomarker-Specific: - Surface markers (EpCAM, EGFRvIII) enable tumor enrichment (Laulagnier et al., 2004, Tauro et al., 2013) - Crosses BBB (Matsumoto et al., 2017) - Multi-analyte detection (DNA/RNA/protein) (Yu et al., 2021)
Ethical Considerations	Cross-Species Risks: - Residual milk proteins (e.g., casein) may trigger allergies (Zhong et al., 2021, Xiao et al., 2024) - Bovine→human miRNA interactions uncertain (Zhong et al., 2021) Regulatory Gaps: No global contaminant standards (Zhong et al., 2021, Medel-Martinez et al., 2024)	Proven Safety: - Low immunogenicity (19/21 allogeneic trials show no rejection) (Lotfy et al., 2023) - Safe in healthy volunteers (inhaled: 2–16 × 10⁸ particles) (Shi et al., 2021) Risks: Long-term carcinogenicity unknown (Lotfy et al., 2023)	Non-Invasive Advantage: - Avoids biopsy complications (Lokhandwala et al., 2017) - Reduces overdiagnosis (ExoDx™ avoids 27% biopsies) (McKiernan et al., 2016) Implicit Risk: Data privacy unaddressed.

## 4 Interactions of MEVs with the gut microbiome

**FIGURE 4:**
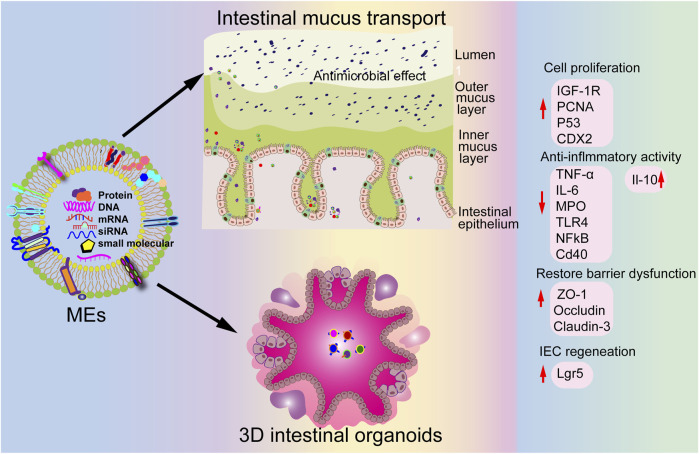
Potential therapeutic effects of MEVs in intestinal diseases.

The gut microbiome comprises more than 100 trillion commensal microorganisms, including bacteria, archaea, fungi, and protozoa, residing in the human gastrointestinal tract (Ley et al., 2006). It plays an essential role in gastrointestinal mucosal function and structure, host protection against pathogens, and maintenance of overall human health (Maynard et al., 2012). Imbalances in the gut microbiota are associated with diseases such as inflammatory bowel disease (IBD), rheumatoid arthritis, diabetes, and obesity in humans (Maynard et al., 2012). Portulaca oleracea L-derived EV-like nanoparticles promote microbiota balance and increase probiotic *Lactobacillus reuteri* proliferation in dextran sulfate sodium salt (DSS)-induced colitis, thereby alleviating colitis (Zhu et al., 2023). Additionally, MEVs play a crucial role in gut microbiota communication, with miR-21, miR-497, and miR-166a MEV cargoes surviving the gastrointestinal tract to target yegH, ptsG, and rpoC, promoting the growth of *Escherichia coli* and *Lactobacillus plantarum in vivo* (Yu et al., 2019). Moreover, Holstein cow MEVs restore *Enterorhabdus* and *unclassified_Bacteroidia* levels while increasing the probiotic *Akkermansia* abundance, contributing to reshaping the gut microbiota in DSS-induced colitis (Figure 4) (Tong et al., 2021). In addition, oral administration of bovine MEVs increases the abundance of several beneficial gut microbes, including *Ruminococcaceae*, *Lachnospiraceae*, and *Akkermansia_muciniphila*, and suppresses pro-inflammatory bacteria levels such as *Proteobacteria* in osteoarthritis, alleviating cartilage degeneration, enhancing matrix synthesis, and reducing cartilage-degrading enzymes (Liu et al., 2023b). These findings suggest that EVs may protect and maintain gut microbiota balance and offer a promising strategy for treating of human diseases.

## 5 Biological activities of MEVs

Studies show that MEVs contain several bioactive components that affect breastfeeding mothers, infants, and even adults who consume milk (Zempleni et al., 2017). MEVs are stable under harsh gastrointestinal conditions, protecting their cargoes for physiological activity (Rahman et al., 2019; Pieters et al., 2015; Izumi et al., 2012). Additionally, they exhibit excellent biocompatibility, being taken up by various cell types, including intestinal epithelial cells (IECs), macrophages, and vascular endothelial cells, and can cross the blood-brain barrier (Del Pozo-Acebo et al., 2021; Wolf et al., 2015; Prasadani et al., 2024; Liu et al., 2024; Zeng et al., 2019). Thus, MEVs can reach various tissues and perform diverse physiological functions.

### 5.1 Intestinal health

Numerous studies show that MEVs are crucial for maintaining intestinal health. Martin et al indicate that MEVs protect IECs against H_2_O_2_-induced oxidative stress (Martin et al., 2018). Moreover, MEVs promote tight-junction proteins ZO-1, claudin-1, and occludin levels *in vitro* and increase goblet cell numbers *in vivo*, helping to alleviate NEC (He S. et al., 2021; Li et al., 2019). Similarly, Chiba et al reveal that MEVs increase ZO-1 expression by inhibiting the expression of key cellular stress gene REDD1 *in vitro* (C et al., 2023). Additionally, MEVs enhance intestinal epithelial cell viability and promote their growth (Hock et al., 2017).

### 5.2 Bone and muscle health

Bone health relies on the balance between osteoblasts (cells that form bone) and osteoclasts (cells that resorb bone). Disruptions in this balance can lead to bone diseases such as osteoporosis. Previous studies indicate that MEVs promote osteogenesis and inhibit osteoclastogenesis, enhancing osteoblast proliferation and differentiation *in vitro* and *in vivo* (Yun et al., 2020). These findings suggest that MEVs may stimulate bone formation, prevent osteoporosis (Yun et al., 2020), and inhibit osteoclast differentiation, thereby reducing bone resorption (Kim et al., 2023; Melnik et al., 2021). These findings suggest that MEVs may play a vital role in maintaining bone density and strength by modulating the activity of bone-forming and bone-resorbing cells (Kim et al., 2023; Melnik et al., 2021). These findings suggest that MEVs may play a vital role in maintaining bone density and strength by modulating the activity of bone-forming and bone-resorbing cells (Kim et al., 2023; Melnik et al., 2021).

Muscle maintenance and growth depend on a balance between protein synthesis and degradation. MEVs support muscle health by promoting myogenesis and tissue formation, particularly in conditions such as sarcopenia and age-related muscle loss. MEVs contain growth factors and proteins that stimulate muscle protein synthesis, inhibit its degradation, and promote muscle hypertrophy while preventing muscle wasting (Parry et al., 2019). In addition, MEVs enhance muscle cell proliferation and development by activating the AKT/mTOR pathway and myogenic regulatory factors (Meng et al., 2023). Therefore, MEVs may offer a novel dietary approach to combat muscle degeneration and support overall muscle health.

### 5.3 Anti-inflammatory and antioxidant activities

Chronic inflammation contributes to the development of several diseases, such as IBD (Rubin et al., 2012; Fernandes et al., 2024; Yan and Shao, 2024). MEVs may help reduce inflammation by inhibiting the expression of pro-inflammatory cytokines such as interleukin-6 and tumor necrosis factor-alpha, thereby alleviating inflammatory response (Cui et al., 2023). Additionally, MEVs reduce pro-inflammatory cytokine levels and attenuate LPS-induced inflammation in macrophages (Matic and Dia, 2022).

Oxidative stress results from an imbalance between reactive oxygen species (ROS) production and antioxidant defenses, contributing to various chronic diseases (Schieber and Chandel, 2014; Korovesis et al., 2023). MEVs have antioxidant properties owing to their antioxidant substance cargoes, such as superoxide dismutase and glutathione peroxidase, which neutralize ROS and reduce oxidative stress (Feng et al., 2021). Moreover, MEVs decrease ROS production and mitigate oxidative stress by inhibiting NOX2 expression and ROS production (Rashidi et al., 2022). They also activate the Nrf2 pathway, enhancing antioxidant gene expression and cellular defenses against oxidative damage (Wang et al., 2021).

### 5.4 Neuronal development and brain health

MEVs have recently gained attention for their potential role in neuronal development and brain health. They cross the blood-brain barrier, offer neuroprotection, and enhance cognitive function, making them promising for maintaining brain health and treating neurological disorders (Zhou et al., 2022). MEVs promote neural stem cell differentiation and proliferation and support neuronal development by regulating genes critical for neurogenesis and synaptic plasticity (Jiang et al., 2021). MEVs exhibit neuroprotective effects by neutralizing ROS and downregulating pro-inflammatory cytokines (Zempleni et al., 2019). They also support cognitive function and overall brain health by contributing phospholipids and sphingolipids to neuronal membrane integrity and facilitating neurotransmission, which is crucial for cognitive processes (Wang et al., 2024). Additionally, MEVs enhance synaptic plasticity, a key mechanism underlying learning and memory, and regulate synaptic strength by targeting specific synaptic proteins (Wang et al., 2024).

### 5.5 Immunity

Studies show that MEVs play crucial roles in immune regulation by modulating T and B cell differentiation, suppressing inflammatory cytokine production in macrophages (Zhang et al., 2019), boosting macrophage phagocytosis, and promoting dendritic cell maturation, thereby enhancing innate immune responses (He Y. et al., 2021). Furthermore, MEVs carry antigens to immune cells to activate antigen-specific T cells and influence adaptive immunity (Komine-Aizawa et al., 2020). They also promote the proliferation and function of regulatory T cells, exerting a key role in maintaining immune tolerance and preventing autoimmune diseases (Munagala et al., 2016).

### 5.6 Immunogenicity and safety profile of MEVs

MEVs demonstrate inherently low immunogenicity and favorable biocompatibility, positioning them as promising drug delivery vehicles (Zhong et al., 2021; Cui et al., 2023). Critical evidence reveals that MEVs evade significant immune activation across administration routes: repeated intravenous injections in mice (up to 6 mg/kg) induce no systemic anaphylaxis or histamine elevation, while chronic oral dosing in rats (300 μg/kg/day for 21 days) maintains serum IgG/IgM levels comparable to controls. (Xiao et al., 2024). This immunological inertness extends to cellular interactions, where MEVs are efficiently internalized by macrophages at concentrations up to 200 μg/mL without cytotoxicity. Systemic safety assessments further confirm the absence of hepatic/renal toxicity, hematological abnormalities, or pathological changes in major organs (Somiya et al., 2018). Notably, zwitterionic modifications (e.g., DSPE-Hyd-PMPC functionalization) enhance this intrinsic safety by mitigating immunogenicity risks associated with conventional PEGylation (Xiao et al., 2024). Despite these advantages, translational challenges persist, including potential allergenicity from residual milk proteins (e.g., casein) in susceptible populations and species-specific immune response variations that warrant human-relevant validation (Xiao et al., 2024; Somiya et al., 2018). Collectively, the robust safety profile of MEVs supports their clinical translation, though ultra-purification protocols and engineered surface designs remain essential for therapeutic applications.

## 6 Milk EVs in drug delivery

Small molecules, proteins, and oligonucleotides offer promising treatments for various diseases; however, their poor bioavailability, non-targeted accumulation, and instability limit their widespread application. MEVs, with their high bioavailability, non-toxicity, low immunogenicity, and stability, are gaining attention as potential drug carriers.

### 6.1 Oral delivery advantages of MEVs

MEVs exhibit exceptional gastrointestinal stability due to their unique structural composition. The sphingomyelin- and cholesterol-rich phospholipid bilayer membrane confers inherent resistance against gastric acid and digestive enzyme degradation (Munagala et al., 2016; Théry et al., 2009). Experimentally, orally administered MEVs maintain structural integrity while traversing the intestinal barrier and selectively targeting gut cells (Wu et al., 2022; Vashisht et al., 2017). This stability enables effective oral delivery of therapeutic cargo, as evidenced by MEVs-loaded insulin and curcumin retaining bioactivity and significantly enhancing oral bioavailability (Wu et al., 2022; Carobolante et al., 2020). MEVs further demonstrate natural tropism for intestinal tissues through surface proteins (e.g., integrins), facilitating mucus penetration and cellular uptake (Warren et al., 2021). Critically, MEVs exhibit no systemic toxicity, with distribution studies confirming prolonged GI retention after oral administration *versus* hepatic/renal accumulation following intravenous injection (Samuel et al., 2021; Tong et al., 2021).

### 6.2 Significant advances in therapeutic delivery

Substantial progress has been achieved in utilizing MEVs as nanocarriers for chemotherapeutic agents and nucleic acid-based therapies. Since 2016, bovine MEVs have been successfully engineered to encapsulate diverse chemotherapeutics including curcumin, withaferin A (WFA), anthocyanins, paclitaxel (PAC), and doxorubicin. (Vashisht et al., 2017; Warren et al., 2021; Agrawal et al., 2017; Luo et al., 2021).Critically, orally delivered MEVs exhibit exceptional resistance to digestive degradation and enhance intestinal permeability—validated in Caco-2 monolayer models. (Vashisht et al., 2017). These vesicles significantly improve drug stability; for instance, curcumin-loaded MEVs demonstrate markedly higher stability in PBS compared to free drug formulations (Vashisht et al., 2017). *In vivo* efficacy is evidenced by WFA-loaded MEVs significantly suppressing lung tumor xenograft growth (Munagala et al., 2016), while PAC-loaded MEVs reduce hepatorenal/systemic toxicity and immunogenicity relative to intravenous administration (Agrawal et al., 2017).

Beyond small-molecule delivery, MEVs effectively transport nucleic acid therapeutics. β-Catenin siRNA loaded via lipofection mediates potent gene knockdown *in vitro versus* scrambled controls (Aqil et al., 2019), while *hsa-miR148a-3p*-enriched MEVs achieve functional delivery in HepG2 and Caco-2 cells, with microarray analyses confirming their utility as miRNA nanocarriers (Del Pozo-Acebo et al., 2021).

### 6.3 Cargo loading

Munagala et al first reported that MEVs, as drug delivery systems, load small drug molecule compounds, including curcumin, withaferin, anthocyanidins, paclitaxel (PAC), and docetaxel, targeting lung and breast cancer cells while enhancing anticancer and anti-inflammatory effects (Munagala et al., 2016). MEVs containing isobavachalcone and polymyxin B effectively combat pathogenic bacteria (Xu et al., 2024), eliminating 99% of multidrug-resistant bacterial pathogens and nearly 100% microbial inhibition in animal models (Xu et al., 2024). Resveratrol (RSV), a natural polyphenolic phytoalexin, exhibits antidiabetic, anti-inflammatory, anticancer, wound healing, and antioxidant effects; however, its poor solubility and stability limit its clinical application (Summerlin et al., 2015; Monika and Sardana, 2017). MEVs loaded with RSV enhance its oral bioavailability and effectively reduce inflammation in experimental colitis (Esfahani et al., 2024). miRNAs, endogenous small non-coding RNA molecules, regulate gene expression post-transcriptionally (Bartel, 2009). and offer potential avenues for treating human diseases (Kazemi Shariat Panahi et al., 2024). However, instability and rapid degradation present challenges (Kazemi Shariat Panahi et al., 2024). Meng et al report that MEVs containing miR-146a (MEVs-miR-146a) suppress myocardial tissue apoptosis reduce inflammatory factor expression, and improve cardiac function by inhibiting the IRAK1/TRAF6/NF-κB signaling pathway after myocardial ischemia-reperfusion injury (MIRI), suggesting a promising strategy for MIRI treatment (Meng et al., 2024). MEVs can deliver oral chemotherapeutic drugs, enhancing their efficacy and reducing toxicity. For instance, MEVs carrying PAC significantly reduced liver, renal, and systemic toxicity while inhibiting the growth of human lung tumor xenografts in nude mice, compared to PAC treatment alone (Agrawal et al., 2017). Therefore, MEVs are promising natural drug vehicles for proteins, drugs, and nucleic acids in disease treatment.

### 6.4 MEV delivery methods

Various strategies, including co-incubation, electroporation, and freeze-thaw cycles, have been used recently to load therapeutic molecules into MEVs. Co-incubation, the simplest and most effective passive drug-loading strategy, allows the loading of photosensitizer chlorin e6, which is crucial for precision treatment of deep solid tumors (Guo et al., 2023). Moreover, drugs such as PAC, curcumin, and docetaxel can be loaded through co-incubation in an appropriate buffer. However, co-incubation has the disadvantage of low load efficiency.

Electroporation utilizes electrical current to disrupt the EV phospholipid bilayer, forming temporary pores through which small molecules can enter. After this process, the membrane integrity of MEVs is restored. Electroporation is generally used to load siRNAs or miRNAs, such as miR-146a and miR-31-5p (Warren et al., 2021; Meng et al., 2024; Yan et al., 2022). However, electroporation may cause membrane instability, RNA aggregation, and low loading efficiency (Luan et al., 2017). This method often causes MEV aggregation and has low loading efficiency (S et al., 2016).

**FIGURE 5:**
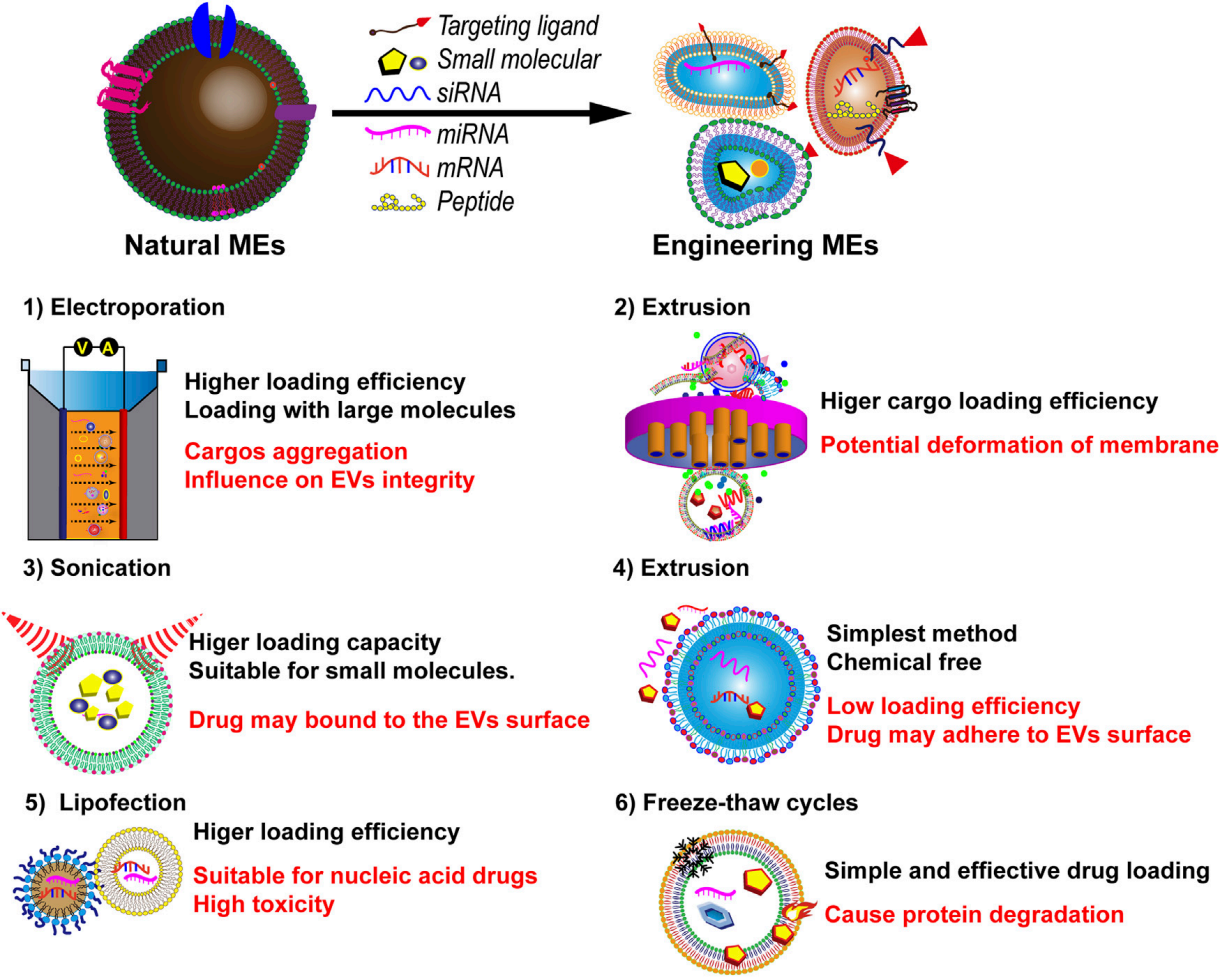
Various current techniques for loading therapeutics into MEVs, and a comparison of their advantages and disadvantages.

Mechanical sonication involves using shear force to disrupt MEV membrane integrity, allowing drug molecules to diffuse into the MEVs during membrane deformation (Luan et al., 2017). Sonication is primarily used for loading proteins and hydrophobic drugs (Tian et al., 2023c). This method has a better loading efficiency than that of other methods (Tian et al., 2023c). However, its disadvantages include time wastage and MEV degradation (Luan et al., 2017). Figure 5 shows the exogenous method for loading various therapeutics into MEVs.

### 6.5 Targeted modification of MEVs

MEVs exhibit considerable promise as oral drug carriers owing to their cross-species tolerance and capacity to traverse gastrointestinal barriers (Zhong et al., 2021). Their evolutionarily conserved surface proteins (e.g., CD9, CD63) facilitate uptake by human intestinal cells, enabling therapeutic efficacy in preclinical models (Kalluri and LeBleu, 2020). However, inherent species disparities may introduce functional variations: differential affinities of bovine exosomal ligands for human receptors could impact cellular internalization, while interspecies divergence in miRNA-mRNA interactions might attenuate regulatory functions (Zhong et al., 2021). Biodistribution patterns—characterized by GI retention after oral administration *versus* hepatic/renal accumulation following intravenous delivery—may exhibit cross-species variability due to distinct immune clearance (Agrawal et al., 2017; Jang et al., 2021). To overcome these limitations and further enhance targeting specificity, significant advances have been made in bioengineering MEVs. The core technologies primarily involve ligand functionalization employing the versatile post-insertion technique. This strategy entails pre-conjugating the targeting molecule (e.g., folic acid (FA), hyaluronic acid (HA), targeting peptides, or antibodies) to a phospholipid anchor molecule, such as DSPE-PEG2000 or phosphatidylethanolamine (PE), followed by its hydrophobic insertion into the MEVs’ lipid bilayer. This achieves a mild, efficient, and non-destructive modification (Cui et al., 2023; Jang et al., 2023).

Functionalization of MEVs with tumor-specific ligands has demonstrated substantial improvements in active targeting. For instance, FA modification leverages the overexpression of folate receptors on certain cancer cells. Orally administered MEVs co-loaded with wheat germ agglutinin (WGA) and FA (Exo-WGA-FA) significantly increased the tumor suppression rate from 50% to 74% (*p* = 0.016) in a lung cancer model, mediated by FA receptor-specific internalization (Munagala et al., 2016). Similarly, HA coating utilizes the binding between HA and the CD44 receptor overexpressed on tumor cells. HA-DSPE-PEG2000 conjugates, formed via amide condensation (validated by NMR and FTIR), are anchored onto the MEVs surface, significantly enhancing the enrichment and therapeutic efficacy of payloads like miR-204 or doxorubicin within tumor tissues (Cui et al., 2023; Cui et al., 2022). Furthermore, peptide display (e.g., the tumor-penetrating peptide iRGD), antibody conjugation (targeting ubiquitous EV membrane proteins like CD63/CD9 or disease-specific antigens), and aptamer modification (e.g., EpCAM aptamer) have also been effectively employed to reprogram the tropism of MEVs towards specific pathological sites (Zhong et al., 2021).

Collectively, these engineering strategies not only improve the accumulation of MEVs at disease sites but also enhance drug delivery efficiency through receptor-mediated endocytosis, while largely preserving their inherent low immunogenicity. They establish a robust technical foundation for the clinical translation of targeted therapies based on engineered milk exosomes (Cui et al., 2023; Jang et al., 2023). To address these challenges, engineering strategies such as surface modification with targeting peptides (e.g., iRGD) or hybrid vesicle systems represent viable approaches to bridge species-specific gaps, as discussed below (Carobolante et al., 2020).

Recent modifications to MEV surfaces enhance drug delivery targeting. While folate receptors are minimally expressed in normal tissues, they are overexpressed in some cancer cells, such as non-small cell lung cancer cells and lung adenocarcinoma cells (Shi et al., 2015; Kandimalla et al., 2021b). Therefore, folic acid-conjugated MEVs can be used to precisely target tumor sites in clinical applications. For instance, folic acid-modified MEVs loaded with apherin A and PAC significantly suppress tumor cell growth compared to that of non-functionalized surface MEVs while reducing system toxicity (Munagala et al., 2016; Kandimalla et al., 2021b). The hyaluronic acid receptor CD44 is significantly overexpressed in pancreatic, lung, ovarian, and breast cancers (Naor et al., 2002). Hyaluronic acid-modified MEVs loaded with doxorubicin specifically target CD44-overexpressing cancer cells, enhancing anticancer sensitivity (Li et al., 2020). Additionally, the slightly acidic pH (6.5–7.4) of many solid tumors offers an alternative target for clinical treatment (Counihan et al., 2018). In such environments, the imine bond, sensitive to pH below 6.8, degrades rapidly (Liu et al., 2019). This property enables hydrazone-bound chemotherapeutic drugs conjugated to MEV membranes to release drugs at tumor sites under acidic conditions (Zhang et al., 2020).

Commercial translation of this technology is exemplified by PureTech Health (founded 2001), which leverages MEVs within its preclinical Discovery platform for oral delivery of biologics (e.g., antisense oligonucleotides) and complex small molecules targeting rheumatoid arthritis, diabetes, autoimmune disorders, and oncology (Munagala et al., 2016; Agrawal et al., 2017; Aqil et al., 2019). Key translational advantages include low-cost scalability through abundant milk sources and reduced immunogenicity inherent to bovine-derived vesicles (Munagala et al., 2016; Agrawal et al., 2017; Aqil et al., 2019).

## 7 Conclusion and future perspectives

This study reviewed the characteristics, isolation, purification, and biological activities of MEVs. Notably, MEVs possess unique advantages of stability, low immunogenicity, biocompatibility, and excellent biofilm penetration. As drug carriers, MEVs offer a promising strategy for treating diseases such as IBD and cancer. Furthermore, their ability to undergo specific modifications significantly enhances therapeutic efficacy and reduces side effects, especially in cancer treatment.

However, the clinical application of MEVs faces several challenges. First, industrial production remains underdeveloped, as most research is limited to laboratory settings, necessitating further efforts to establish standardized production platforms. Second, few clinical trials have evaluated the pharmacological effects and pharmacokinetics of MEV-based drug delivery, highlighting the need for more robust evidence to support their widespread clinical use. Third, storage conditions significantly impact the quantity, purity, and biological activity of MEVs, yet no consensus exists on optimal storage practices to preserve their integrity and functionality, warranting further functional studies. Fourth, the low drug-loading efficacy of MEVs necessitates the development of more effective methods.

MEVs represent a promising natural nanocarrier for drug delivery and therapeutic applications. Their stability, biocompatibility, and low immunogenicity make them ideal candidates for targeted therapies, particularly in oral delivery and disease treatment. However, challenges in industrial production and clinical application, such as inconsistent isolation methods, safety concerns, and limited clinical data, must be addressed. Future research should focus on optimizing isolation and purification techniques, developing efficient oral delivery systems, and enhancing the targeting and stability of MEVs through functional modifications. Large-scale clinical trials and comprehensive safety assessments will be critical for translating MEVs from the laboratory to clinical practice. Through interdisciplinary collaboration and technological innovation, MEVs hold the potential to revolutionize disease treatment and health management, offering novel solutions in both nutritional and medical fields.

## 8 Plain language summary

• Mammalian milk is a rich source of extracellular vesicles (EVs).
• Milk-derived EVs are used to load small molecules for therapeutic purposes.
• MEVs with targeted ligand function are used for tissue/organ-targeted therapy.

## Abbreviations

MEVs, Milk-derived extracellular vesicles; EVs, extracellular vesicles; miRNA, microRNA; ESCRT, endosomal sorting complex required for transport; PBMC, peripheral blood monoculear cell; ICAM-1, intercellular cell adhesion molecule-1; PC, phosphatidylcholine; PE, phosphatidylethanolamine; PS, phosphatidylserine; NECv, necrotizing enterocolitis; NSCs, neural stem cells; IEC-6, intestinal epithelial cell-6; LPS, Lipopolysaccharides; SEC, size exclusion chromatography; IBD, inflammatory bowel disease; PELNs, Portulaca oleracea L-derived EV-like nanoparticles; DSS, dextran sulfate sodium salt; OA, osteoarthritis; IECs, intestinal epithelial cells; NEC, necrotizing enterocolitis; REDD1, regulated in development and DNA damage response 1; MRFs, myogenic regulatory factors; IL-6, interleukin-6; LPS, lipopolysaccharide; ROS, reactive oxygen species; SOD, superoxide dismutase; GPx, glutathione peroxidase; Tregs, regulatory T cells; IS, isobavachalcone; PB, polymyxin B; IP-MEVs, MEVs comprising isobavachalcone and polymyxin B; MDR, multidrug-resistant; RSV, Resveratrol; MEVs-miR-146a, MEVs containing miR-146a; MIRI, myocardial ischemia-reperfusion injury; PAC, paclitaxel.
